# Ablation Techniques for Mahaim Fiber Tachycardia

**Published:** 2009-03-15

**Authors:** Shomu Bohora, Jaganmohan Tharakan

**Affiliations:** 1 Consultant, Baroda Heart Institute and Research Centre, Vadodara, India; 2 Professor and Head of Department of Cardiology, Sree Chitra Tirunal Institute for Medical Sciences and Technology, Trivandrum, India

**Keywords:** Mahaim Fiber, Radiofrequency ablation, Arrhythmia

## Abstract

Mahaim fiber exhibits atrio-ventricular node like properties and generally is localized at the lateral aspect of the tricuspid annulus. Of the varying methods for localization, ablation at the site of Mahaim potential is the most accepted and successful method. Radiofrequency ablation of Mahaim fiber has high success rates.

## Introduction

 Mahaim fiber tachycardia is not an uncommon cause of wide QRS tachycardia in young having no structural heart disease. Mahaim and Winston were the first to describe Mahaim fiber, as a tract, which connects the atrio-ventricular (AV) node to the ventricular myocardium [1]. It was initially believed that Mahaim accessory pathways represent nodofascicular or nodoventricular connections. In 1978 Becker et al  [2] found specialized fibers present at the lateral tricuspid annulus and coursing through the right ventricle mimicking a second AV node. Later, electrophysiology (EP) study, catheter and surgical ablation established the fact that atriofascicular fibers connecting the lateral tricuspid annulus generally to right bundle, are predominantly responsible for Mahaim conduction  [3-12].

 Mahaim Fiber conducts only antegrade and has decremental conduction property. Due to its AV node like EP property it is also known as an ectopic AV node [2,8]. Mahaim fiber conduction is characterized by gradual increase in the AV interval simultaneous with the development of LBBB and shortening of the His-ventricular (HV) interval in response to atrial overdrive pacing [3,4]. Tachycardia shows a left bundle branch block (LBBB) morphology, with left axis deviation (LAD) on electrocardiogram (ECG) as the antegrade limb is formed by the Mahaim fiber and the AV node forms the retrograde limb of the circuit. The electrophysiological properties of antegrade only, with decremental conduction, are well elucidated by many previous studies [4,5,7,8].

## Localization of the Mahaim fiber at the tricuspid annulus

Anatomically Mahaim Fiber is located at the lateral tricuspid annulus in most instances. Location of the pathway in occasional cases may be variable along the tricuspid annulus especially when associated with Ebstein's anomaly which is a common associated structural anomaly along with Mahaim fiber tachycardia [10,11,14-20]. Left side accessory pathway with characteristics of Mahaim Fiber has rarely been described [21]. Faizel et al report such a rare case in this issue of the journal [22]. 

## Methods for mapping and radiofrequency (RF) ablation

Once Mahaim fiber related tachycardia is confirmed by EP study, mapping is generally performed along the lateral tricuspid annulus, which is the most common localization site. Because of their conduction properties and anatomic location, it is difficult to map both the proximal (atrial) and distal (ventricular) insertion of Mahaim fibers. Mahaim fibers do not conduct retrogradely so the proximal insertion cannot be identified by ventricular pacing. They tend to have generally a long course and often show extensive arborization over a wide area of ventricular muscle making ablation at the ventricular insertion site difficult [15]. There are several reports on ablation of Mahaim accessory pathway with a very high success rate [7,10,11,13-20]. Different strategies can be adapted for successful localization of the pathway for ablation.

**Figure 1:**
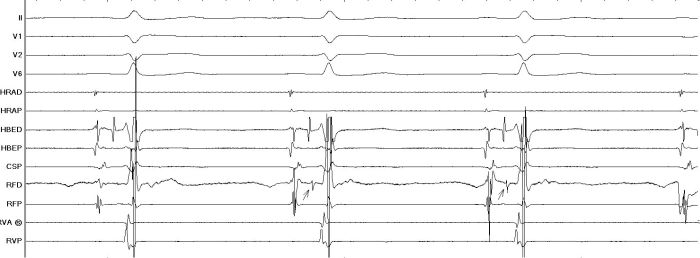
Mahaim fiber potential as marked with arrows in the RF Distal (RFD) channel shows a sharp potential similar to a His recording. (HBED)

 Mahaim (M) potentials (Figure 1) appear as discrete sharp potential lying between the atrial and ventricular electrograms recorded at the tricuspid annulus. The interval between the M potential and V always remains constant during atrial pacing at different cycle lengths. The potential may be as large as the His bundle potential or small and narrow with low amplitude. A Mahaim potential will be recorded only in very close proximity to the atrial insertion of the accessory pathway and, therefore, is likely to be a good predictor of a successful ablation site as described by various studies [7,11,15-18]. Several authors [11,14-18](11,14-18) have shown a success of 100% while McClelland et al [7] reported a success of 88% for ablations guided by M potentials. Recurrence has rarely been reported once successful ablation has been done by this method [18,20].

Mechanical trauma induced loss of conduction via Mahaim fiber has also been described. Mahaim fiber is located very close to the endocardium and thus catheter movement related mechanical trauma resulting transient loss of conduction is not uncommon. Mapping and RF ablation guided by this method has been useful [13,20]. Unintentional mechanical trauma during catheter positioning sometimes result in transient abolition of conduction through the pathway for minutes to hours [10,17], which may hamper successful localization and ablation thereafter and may be responsible for unsuccessful procedure outcome. Cappato et al [13] showed a very high immediate success rate with this method, but there were high recurrences at follow up.

Activation mapping of the earliest local ventricular potential targeting distal branches is a time consuming task. It is possible to ablate some of them using this technique but mostly a complete elimination is very unlikely. Some patients with atriofascicular pathway who underwent ablation at the distal insertion had developed a pro-arrhythmic response with facilitation of antidromic tachycardia occurrence due to slow conduction induced by radiofrequency ablation [7,10]. Also ablation of the right bundle branch within the ventricle can result in lengthening of the tachycardia circuit and more incessant tachycardia. Because of these problems, ablation is usually aimed at the proximal (atrial) insertion of the pathway. In some cases, however, mapping and subsequent ablation of the ventricular insertion has been achieved leading to successful clinical outcome as described by the case report by Valentino et al in this issue of the journal [23].

 Shortest interval from stimulus to ventricular activation is defined as the shortest interval between the pacing sites at different sites at the RA side of the tricuspid annulus to the earliest preexcited QRS. The result of RF ablation at the site of the shortest interval has remained inconsistent as seen in various studies and varies from 0 to 100% [10,17-20].  This method is time and labour intensive and very inaccurate because it is difficult to stimulate from many sites at the same distance from the annulus. However in the absence of a proper M potential, this method is helpful in localization of the Mahaim fiber.

Similar to the previous technique, atrial extra-stimulus mapping during antidromic tachycardia is a time consuming method for finding an atrial site where the longest coupled premature extra-stimulus causes resetting. The site in the atrial annulus with the least interposing tissue separating it from the accessory pathway proximal insertion is obtained which is a suitable site for attempting ablation.

Electro-anatomic mapping [19,24] can be helpful in cases where accessory pathway potential cannot be found and there has been a failed attempt with above conventional techniques. The earliest annular or ventricular activation can be known and the sites can be tagged as the probable sites for ablation. This technique allows the operator to reach the tagged sites and further ablations can be attempted in and around the area. However success rates have not been encouraging [19].

## Mahaim Automatic Tachycardia

**Figure 2:**
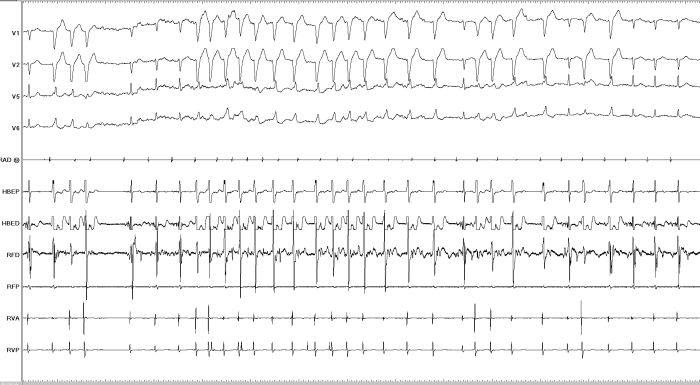
Radiofrequency ablation at the site of M potential is causing accelerated Mahaim Fiber automatic rhythm.

 "Mahaim" automatic tachycardia (MAT), is generally brought about during radiofrequency current delivery (Figure 2), though is occasionally seen to occur spontaneously too and is probably due to heat-related automaticity of nodal-like tissue (in a similar fashion to junctional rhythm that arises during slow A-V nodal pathway ablation). Accelerated automatic beats during RF ablation have been considered as a marker of successful result [7,14,18-20].

## Important practical points with regard to ablation

Ablation catheter should be moved slowly and carefully along the annulus, avoiding bumps on the tissue so as to avoid a mechanical trauma and accidental loss of preexcitation, for which long sheaths can be used for improving stability. Ablation, if possible, should be done during atrial pacing. Stability is improved during atrial pacing as compared with ablation during antidromic tachycardia when catheter is likely to move with tachycardia termination. MAT can also cause catheter displacement and therefore atrial pacing during ablation is beneficial. Also radiofrequency current applied during atrial pacing increases preexcitation and hence make it easier to assess conduction block.

In occasional patients in spite of all attempts to localize the pathway, ablation may not be successful [19,20]. This may be due to presence of accessory pathway at uncommon sites including an epicardial location.

 Coexistence of other tachycardias, especially atrioventricular nodal reentrant tachycardia (association of up to 10-40%) and WPW syndrome due to Kent bundle has been described in literature [7,14,16-20]. Coexisting atrial flutter has also been described [20].  Therefore after successful ablation, programmed electrical stimulation to exclude the presence of other arrhythmias is a must.

Summarizing, Mahaim fiber exhibits AV node like properties and generally is localized at the lateral aspect of the tricuspid annulus. Successful ablation of the accessory pathway is achieved using varying methods, the most successful being the site of the M Potential. MAT is generally obtained along with a successful ablation. Radiofrequency ablation of Mahaim Fiber has a high success rate.
